# Effectiveness of Space Maintainers in Pediatric Patients: A Systematic Review and Meta-Analysis

**DOI:** 10.3390/dj13010032

**Published:** 2025-01-14

**Authors:** MDolores Casaña-Ruiz, Juan Ignacio Aura-Tormos, Laura Marques-Martinez, Esther Garcia-Miralles, Marcelino Perez-Bermejo

**Affiliations:** 1 Dentistry Department, Faculty of Medicine and Health Sciences, Catholic University of Valencia San Vicente Mártir, 46001 Valencia, Spain; 2 SONEV Research Group, School of Medicine and Health Sciences, Catholic University of Valencia, 46001 Valencia, Spain

**Keywords:** space maintainer, pediatric patients, fixed space maintainer, unilateral space maintainer, bilateral space maintainer

## Abstract

**Background:** Evaluating the effectiveness and survival rates of space maintainers is crucial for optimal pediatric dental care. The present study’s goal is to evaluate the survival rate of different types of space maintainers—removable, fixed with a metal base, or fixed with a resin base—indicated for children as a consequence of premature loss of primary teeth. **Methods:** A systematic review was conducted in accordance with PRISMA recommendations and was previously registered in PROSPERO under registration number: CRD42024534183. The search was performed in five different databases PubMed, EMBASE, Scopus, Cochrane, and Web of Science. This was supplemented by a manual database search. From the initial electronic search of the five databases, 72 articles were identified after removing duplicates. After reading the titles and abstracts, 46 articles were excluded, leaving a total of 26 studies. Upon reading the full texts, 15 were excluded for not meeting the inclusion criteria, resulting in a total of 11 articles included in the systematic review. **Results:** The total number of patients treated in the studies was 479, with four observational studies and seven randomized clinical trials. Fixed space maintainers, such as band and loop (BL) or lingual arch (LA), are preferred due to their long-term stability and lower risk of loss. In contrast, removable maintainers, such as Hawley plates, are suitable temporarily but require more patient cooperation and may be less tolerated. **Conclusions:** Space maintainers help prevent the migration of adjacent teeth and the need for more invasive orthodontic treatments in the future. Additionally, they contribute to adequate masticatory function and speech development.

## 1. Introduction

Premature loss of primary teeth refers to the early loss of a primary tooth from the dental arch, occurring at least 12 months before the expected eruption of its corresponding permanent tooth. This condition takes into account the natural variations in the exfoliation sequences of primary teeth and the associated range of timing [1].

The early loss of primary molars is often a result of early extraction, typically due to extensive caries or unsuccessful pulp therapy. Such premature loss can lead to the migration of adjacent teeth, causing significant space loss, crowding, and potential impaction or displacement of permanent teeth. This, in turn, may necessitate complex and prolonged orthodontic interventions [2].

In pediatric dentistry, one of the most significant challenges is managing space loss effectively. During the transition from primary to permanent dentition, it is essential to maintain adequate space to ensure the development of functional, aesthetic, and stable occlusion. Any disruption in this process can severely affect the final alignment and occlusion of the permanent teeth [3].

Fortunately, many of these complications can be prevented or minimized, even when the premature loss of teeth is unavoidable, if proper planning is carried out and space maintenance is performed during the mixed dentition phase [4].

Several types of space maintainers are available, depending on factors such as the child’s age, dental arch development, and cooperation level. These appliances are crucial for guiding the alignment and eruption of permanent teeth [5].

**Figure 1:**
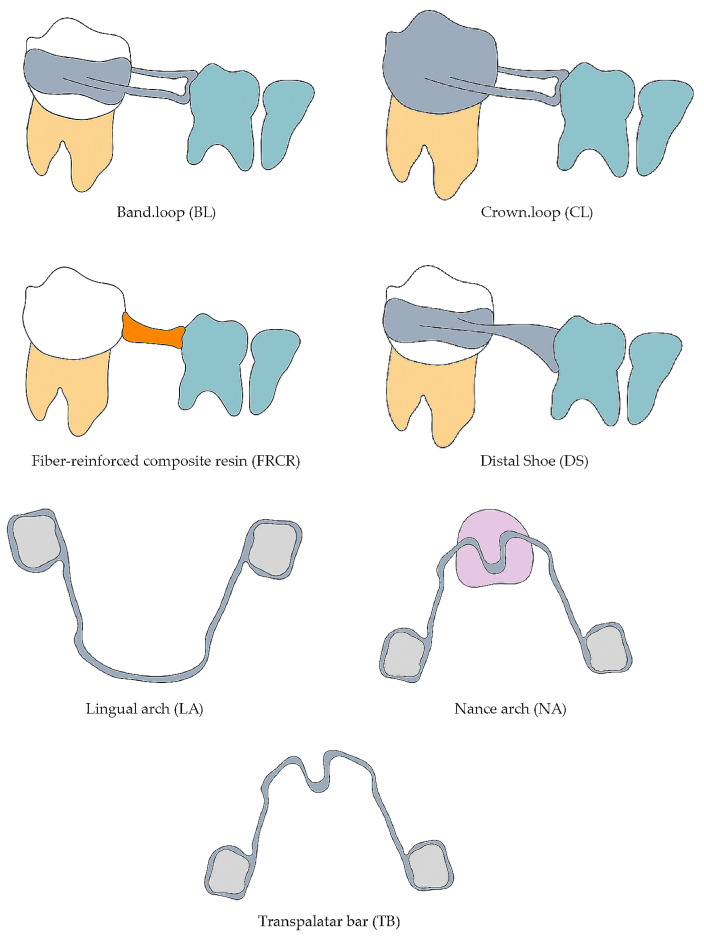
Fixed space maintainers.

Space maintainers can be either fixed or removable, and unilateral or bilateral. Commonly used fixed unilateral space maintainers include the band–loop (BL), crown and loop (CL), direct bonding (DB), fiber-reinforced composite resin (FRCR), and distal shoe (DS). Fixed bilateral space maintainers, such as the lingual arch (LA), Nance arch (NA), and transpalatal bar (TB), are also frequently employed [6] (see Figure 1).

**Figure 2:**
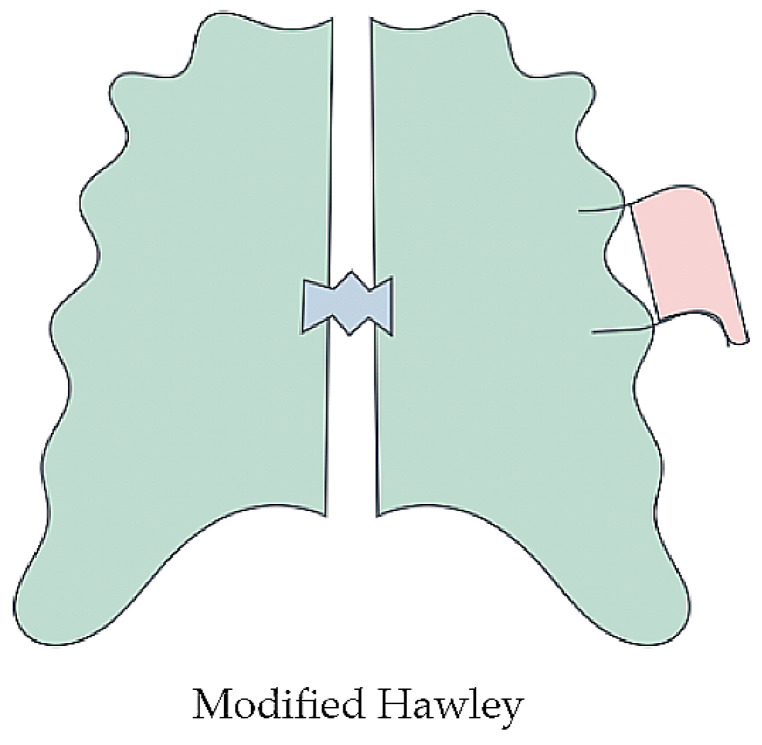
Removable space maintainer.

For removable space maintainers, Hawley appliances are commonly used. These have been adapted with an acrylic base and retention hooks, specifically Adams clasps for permanent molars or second primary molars, along with arrow-shaped clasps [7] (see Figure 2).

Removable space maintainers have some limitations, including poor retention, reduced patient comfort, and a higher likelihood of displacement, which makes fixed space maintainers more suitable for extended use. On the other hand, while fixed space maintainers are generally well-tolerated and durable, they require annual removal for inspection, cleaning, and the application of fluoride treatment, which is not necessary for removable appliances [1].

As a result, resin-bonded fixed space maintainers have become increasingly popular due to their ease of attachment, simpler fabrication, and enhanced patient comfort. Moreover, they eliminate the need for yearly inspections required by other fixed appliances, making them a practical alternative to traditional fixed space maintainers [8,9].

The purpose of this study is to assess the survival rates of different types of space maintainers—removable, fixed with a metal base, and fixed with a resin base—used in children following the premature loss of primary teeth.

## 2. Materials and Methods

### 2.1. Protocol and Registration

A systematic review of the literature was carried out in accordance with the PRISMA 2020 recommendations (PRISMA: Preferred Reporting Items for Systematic Reviews and Meta-Analyses; the PRISMA 2020 statement: an updated guideline for reporting systematic reviews). The present systematic review was previously registered in PROSPERO under registration number CRD42024534183.

### 2.2. Information Sources

A comprehensive electronic search was conducted in the Medline (PubMed), Excerpta Medica (EMBASE), Scopus, Web of Science, and Cochrane databases to identify potentially relevant studies regardless of language. In specific cases, authors of articles were contacted via email to request any necessary information. A manual search of the references of included studies was performed to identify any articles that might meet the inclusion criteria and were not found in the databases. The search was last updated in March 2024.

PICO question: Are dental space maintainers effective in children?

P: Pediatric patients (≤12 years old) with deciduous or mixed dentition.

I: Space maintainers

C: Comparator not applicable

O: Survival rate.

### 2.3. Search Strategy

All keywords were selected based on medical subject headings (MeSH) and non-MeSH terms. The main keywords included were:

- Child* OR Paediatric OR “Pediatric patients” OR “primary dentition” OR “deciduous dentition” OR “mixed dentition” OR “transition dentition”;
- “Space maintainer*” OR “fixed space maintainer*” OR “unilateral space maintainer” OR “bilateral space maintainer”;
- “Failure-rates” OR “success-rates”.

The search equation was: ((ALL = (“Failure-rates” OR “success-rates”)) AND ALL = (“space maintainer*” OR “fixed space maintainer*” OR “unilateral space maintainer” OR “bilateral space maintainer”)) AND ALL = (Child* OR Paediatric OR “Pediatric patients” OR “primary dentition” OR “deciduous dentition” OR “mixed dentition” OR “transition dentition”).

### 2.4. Eligibility Criteria

The eligibility criteria of the included studies were determined with the aim of evaluating the clinical effectiveness of the space maintainer.

- Inclusion criteria: Articles and articles in press were included in the study, including randomized clinical trials, longitudinal studies, retrospective and prospective cohort studies, and case-control studies. No restrictions were applied regarding the year of publication or language. The inclusion criteria applied were (1) studies conducted in pediatric patients with premature loss of unilateral or bilateral primary first molars; (2) premature loss of the second primary molar whenever the permanent first molar had erupted unilaterally or bilaterally; and (3) studies conducted in children aged 3–12 years.
- Exclusion criteria: Studies with patients who have non-normal occlusion conditions, such as crossbite, open bite, or deep bite; with patients who have an absence of successor teeth or dental germ; and on space maintainer fabrication methods.

### 2.5. Data Extraction

The search strategy was meticulously designed, considering prior research in the field and their most frequently cited descriptors. To ensure accuracy, a thorough search for duplicates was conducted by cross-referencing the identified references and transferring them to Mendeley’s reference management software. Subsequently, two independent reviewers (MD-CR and L-MM) evaluated the titles and abstracts of all identified articles. In instances of disagreement, a third author (E-GM) was consulted for resolution. Some articles required a full-text review when abstracts lacked sufficient information for assessment. Finally, articles meeting the predetermined criteria were selected for inclusion in the study. Various parameters were established to assess study characteristics, methodology, and results. Authorship and publication year were utilized to differentiate between studies. Data on failure and success rates, along with clinical factors, were recorded. Significant findings and conclusions from each analyzed study were integrated into the outcome variables for comprehensive analysis.

### 2.6. Risk of Bias and Quality Assessment

The studies’ quality was evaluated independently by the same reviewers, employing the NIH quality assessment tool tailored for observational cohort and cross-sectional studies. Any disparities in the quality assessment were resolved through reviewer consensus, with the involvement of a third reviewer if necessary.

### 2.7. Assessment of Quality of Evidence Presented by This Review

Different quality assessments were employed to evaluate the comprehensive quality of evidence for each outcome outlined in this systematic review. This system encompasses five critical domains of assessment: risk of bias, imprecision, inconsistency, indirectness, and publication bias. Studies were subjected to downgrading from a “high quality” rating by one level for significant issues and by two levels for severe issues within these five domains.

### 2.8. Data Synthesis and Statistical Analysis

Meta-analyses on the prevalence of fracture, flexion, and decementation were performed. Study heterogeneity was assessed using Cochran’s Q test and the I2 index, with significant heterogeneity accepted if *p* < 0.05 in the Cochran’s Q test and high heterogeneity if it was greater than 50% [10]. Because of the large heterogeneity, a random-effects meta-analysis was used [11]. Publication bias in the meta-analysis was detected by visual inspection of funnel plots and Egger’s test [12]. Only studies that reported the prevalences analyzed were included in each meta-analysis.

## 3. Results

### 3.1. Study Selection Characteristics

**Figure 3:**
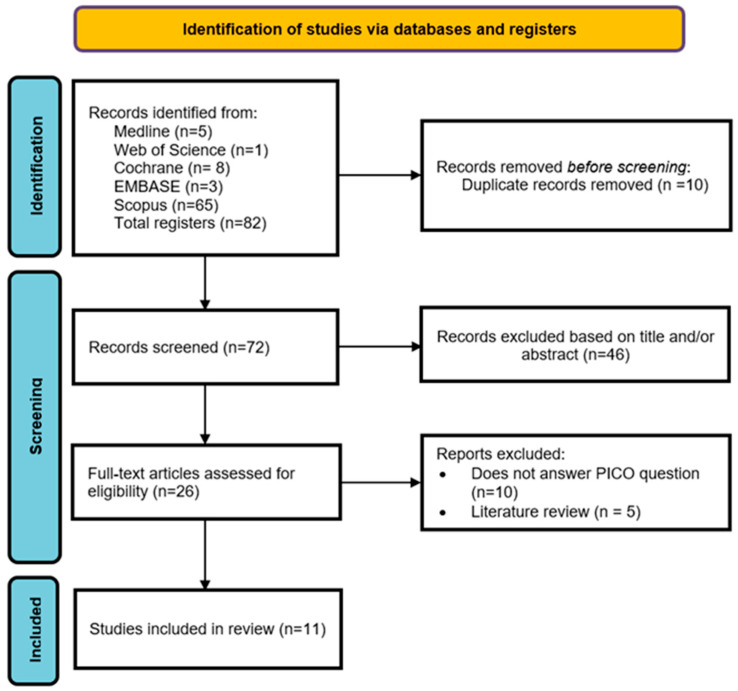
The PRISMA flow diagram.

From the electronic search, a total of 82 results were obtained: Medline (5), Web of Science (1), EMBASE (3), Scopus (65), Cochrane (8). After removing duplicates, 72 studies remained. Upon reviewing the titles and abstracts, 46 were excluded, leaving a total of 26 studies. After reading the full text, 15 were excluded for not meeting the inclusion criteria, leaving a total of 11 articles included in the systematic review. The PRISMA flow diagram provides an overview of the selection process (Figure 3).

### 3.2. Results of Individual Studies

In all included studies, the number of patients ranged from 1513 to 17,314, with a follow-up time ranging from 6 months to 31.6 months. The mean age of patients ranged from 5 to 6.9 years. Three studies [13,14,15] reported the total number of patients by gender, with an accumulated total of 61 boys and 60 girls. All studies, except for Abdin et al. [13], specified that the ages of the patients ranged from 4 to 10 years. The total number of treated patients was 479. Four of the studies [16,17,18,19] were observational, and the remaining seven [14,15,16,20,21,22,23] were randomized clinical trials (RCTs). Regarding the type of space maintainer, fixed space maintainers were used in all studies, among which BL, CL, DS, and FRCR were highlighted, respectively. Only the study by Abdin et al., 2024 [13] compared the effectiveness of removable space maintainers (Table 1).

### 3.3. Quality Assessment

The evaluative process of study quality was carried out independently by identical reviewers, employing the NIH quality assessment tool for observational studies and PEDro for randomized controlled trials (RCTs). Although alternative scales, such as RoB 2.0, are recognized as robust tools for assessing the risk of bias in randomized controlled trials, the PEDro scale was specifically chosen for this study due to its methodological strengths and its enhanced suitability for the particular demands of our research context. Instances of discordance in quality assessment prompted a collaborative resolution among the reviewers, with recourse to consultation with a third reviewer if consensus could not be reached (Table 2 and Table 3).

### 3.4. Qualitative Synthesis

The choice of space maintainer depends on factors such as the patient’s age, dental arch development, and cooperation. Fixed maintainers, such as the band and loop (BL) or lingual arch (LA), are preferred for their long-term stability and lower risk of loss. In contrast, removable maintainers, such as Hawley appliances, are suitable temporarily but require more patient cooperation and may be less tolerated. Proper selection is crucial for optimal dental development and reducing the need for complex orthodontic treatments in the future.

#### Success Rate/Survival Rate

The data provided offer varied insight into the success rates of space maintainers in different cases. It is interesting to note the range of outcomes, from very high [20] to very low success rates [23]. This suggests that there are several variables that can influence the success of space maintainers, such as the specific device design, patient compliance, the dental professional’s skills, and other variables.

### 3.5. Quantitative Synthesis

**Figure 4:**
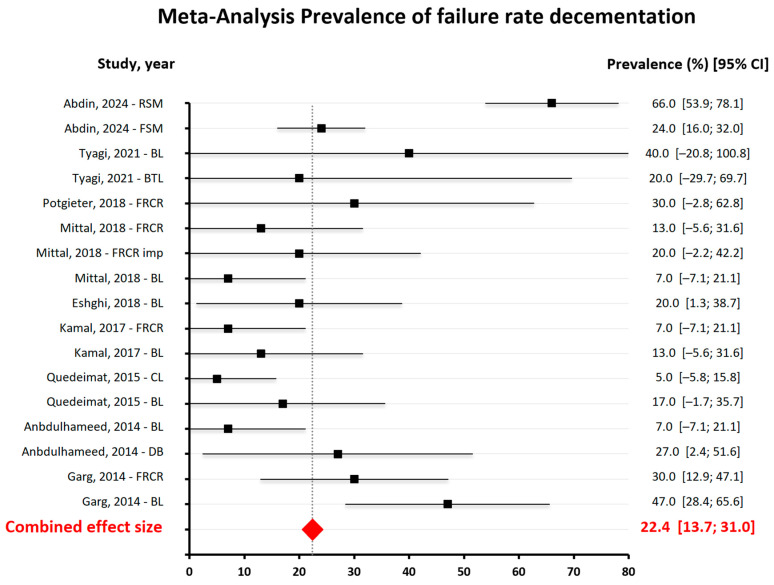
Meta-Analysis findings on the prevalence of the failure rate of decementation [13,14,15,16,18,20,21,22,23].

**Figure 5:**
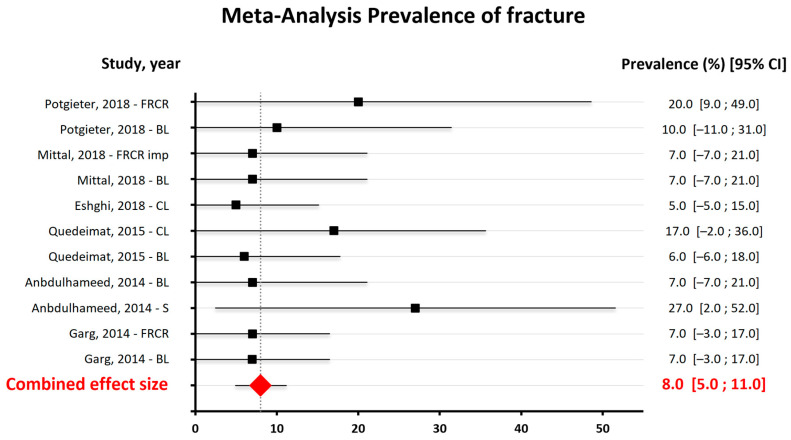
Meta-Analysis findings on the prevalence of fracture [14,15,16,17,18,22,23].

**Figure 6:**
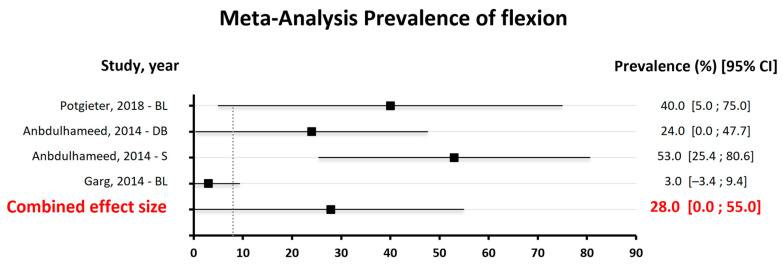
Meta-Analysis findings on the prevalence of flexion [15,18,22].

The results of the quantitative analysis are represented in Figure 4, Figure 5 and Figure 6.

Of the eleven studies selected for the review, only nine were included in the quantitative analysis of the data due to the impossibility of obtaining the necessary information from the other two papers. Three independent analyses were performed: the first related to decementation, the second to fracture and the third to bending. In each of these analyses, all types of space maintainers present in the studies were considered, including band–loop (BL), crown–loop (CL), fiber-reinforced composite resin (FRCR), and distal shoe (DS).

The failure rate in cementation shown in Figure 4 indicates that it varied significantly among studies, but the combined prevalence suggests that this is a relatively common issue, affecting approximately 22.4% of cases. The breadth of confidence intervals varied among studies, reflecting differences in sample sizes and estimation precision [24,25].

The second forest plot, in Figure 5, shows that the use of space maintainers is associated with the prevalence of fractures in children, and the combined effect size is quite robust, with a narrow 95% confidence interval (CI) of [5.0–11.0] [26].

The third forest plot (Figure 6) displays the prevalence of bending, with an effect size of 28.0 and a 95% confidence interval of [0.0–55.0]. Thus, there is an association between bending and the studied outcome, given that the effect size is significantly different from zero [27].

### 3.6. Publication Bias

**Figure 7:**
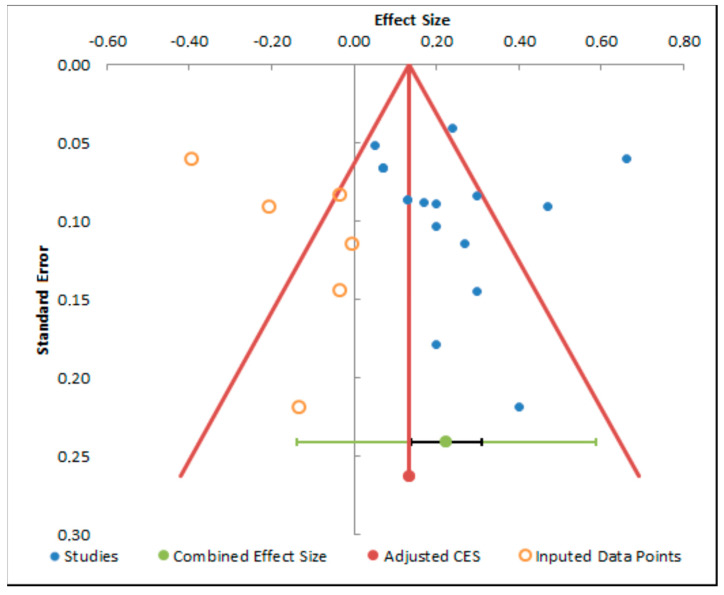
Funnel plot analysis of decementation.

**Figure 8:**
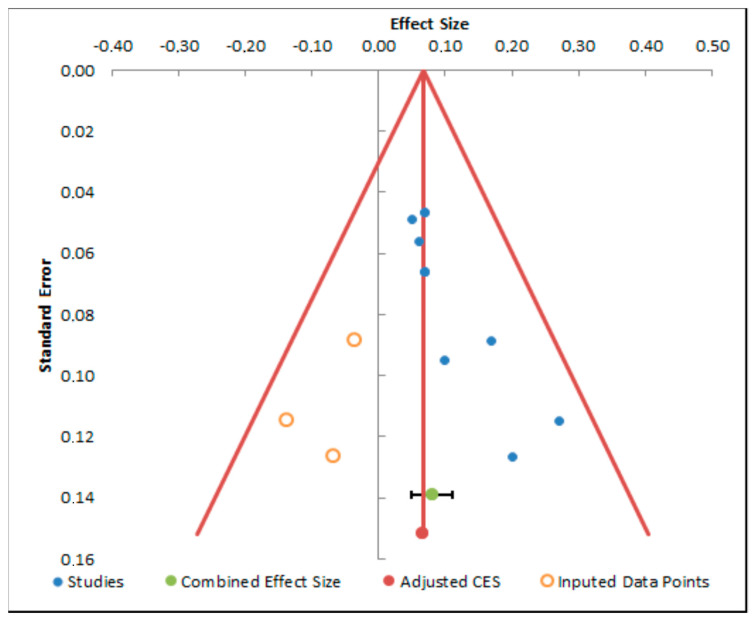
Funnel plot analysis of fracture.

**Figure 9:**
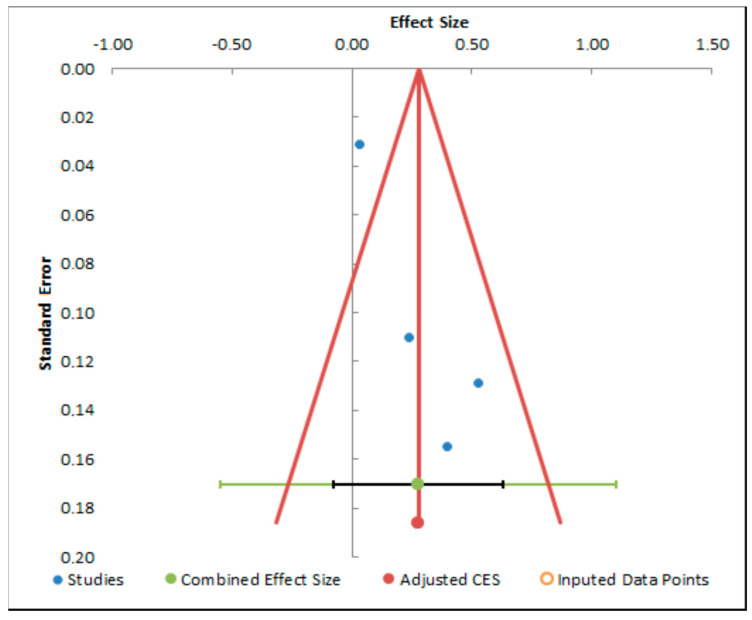
Funnel plot analysis of flexion.

The funnel plot analyses (Figure 7, Figure 8 and Figure 9) display asymmetries, possibly suggestive of publication bias. Incorporating imputed data points assists in rectifying this imbalance, yielding an adjusted estimate of the combined effect size. This adjustment is paramount for a more precise interpretation of the meta-analysis findings and for acknowledging potential limitations stemming from publication bias.

## 4. Discussion

The goal of this systematic review was to assess the clinical effectiveness of space maintainers in dentistry, drawing on observational studies and randomized clinical trials (RCTs). In determining the most effective type of space maintainer, key factors such as longevity and clinical outcomes—including gingival health, plaque accumulation, the condition of supporting teeth, and the ease of appliance fabrication—play a significant role.

Some studies suggest that the premature loss of the first primary molar leads to a reduction in arch length, making the use of space maintainers necessary [13,17,28]. However, other research argues that arch length remains unaffected following the early loss of the first primary molar, implying that space maintainers may not always be required [29]. This difference in opinion highlights the importance of individualized treatment, where the decision to use a space maintainer should depend on the clinician’s judgment and the specific oro-facial characteristics of the patient.

In 2023, Zhao et al. [29] conducted a comprehensive analysis of space changes after the premature loss of the first primary molar. Their meta-analysis identified factors influencing space loss, including age, facial pattern, duration of tooth loss, and molar relationships.

One of the most common causes of failure in fixed space maintainers is debonding. This issue is often linked to the adhesive strength of primary tooth enamel, which is generally weaker than that of permanent teeth. Primary tooth enamel contains prism-free areas that reduce bond strength, negatively impacting resin retention. Additional factors that can compromise adhesion include improper surface preparation, moisture contamination, and issues during the adhesive setting process [30,31].

The survival rates for both metal-based and resin-based space maintainers vary widely, as do survival rates within metal-based maintainers themselves [16,23]. The reported survival rates range from 20% to 86.3%, with follow-up periods between 6 and 18 months. However, there is insufficient evidence to recommend a specific type of fixed space maintainer due to the lack of well-designed studies on this topic.

Some studies, such as those by Abdin et al. [13] and Tahririan et al. [17], report high success rates, with prefabricated maintainers showing a 92% success rate. On the other hand, other studies present more variable results. For instance, Tyagi et al. [21] found a 100% success rate in certain groups, while others had lower success rates.

The variation in success rates can be attributed to numerous factors, including the type of space maintainer used, patient age and cooperation, and the skill level of the dental professional. Moreover, differences in study design, execution, and the definition of “success” across studies may also explain discrepancies in outcomes.

The main limitations of our search were the lack of quality studies with large samples, the homogeneity of studies in terms of design, and the lack of information on certain relevant variables, such as sample selection, allocation, randomization, blinding, and follow-up. The influence of confounding factors, such as age, gender, type of space maintainer, and side(s) of the jaw, which could directly affect the survival rates observed in each study, would therefore also be considered limitations of the review. Since there are no well-designed clinical trials comparing the different types of space maintainers, it is not possible to draw definitive conclusions.

Overall, the analyzed data underscore the importance of carefully selecting the space maintainer type, as well as adequate follow-up to assess space maintainer effectiveness over time. Additionally, they highlight the need for ongoing research to better understand the factors influencing the success of space maintainers and, thus, improve orthodontic treatment outcomes in pediatric patients.

Clinically, it is important to know the different types of space maintainers in order to personalize treatment, although, from the obtained results, the overall satisfaction rates are high.

## 5. Conclusions

Dental space maintainers are highly effective in pediatric dentistry, as they prevent dental misalignments and promote the optimal development of permanent teeth by preserving space following the premature loss of primary teeth. The efficacy of space maintainers depends on selecting the appropriate type of device and carefully considering patient-specific factors. The variability in outcomes underscores the necessity for personalized treatment planning to maximize the benefits of space maintainers and minimize the risk of complications.

## Figures and Tables

**Table 1:** Selected studies and their characteristics.

Study	Type	Sample G/B	Age	Follow-Up (Months)	Maintainer Type	Patients per Group	Decementation	Fracture	Flexion	Survival Rate
Abdin, 2024 [13]	Observational	173	NR	31.6	RSM	61	40/61	NR	NR	21/61
					FSM	112	27/112	NR	NR	85/112
Tyagi, 2021 [21]	Randomized Clinical Trial	20	4–8	9	BL	5	0/5	NR	NR	5/5
					BL	5	2/3	NR	NR	3/5
					BTL	5	1/4	NR	NR	4/5
					CTL	5	0/5	NR	NR	5/5
Tahririan, 2019 [17]	Observational		4–9	9	BTL	-	-	-	-	92%
					Prefabricated					
Potgieter, 2018 [22]	Randomized Clinical Trial	20	4–9	6	FRCR	10	3/10	2/10	0/10	5/10
					BL	10	0/10	1/10	4/10	5/10
Mittal, 2018 [23]	Randomized Clinical Trial	45	6–9	12	FRCR	15	2/15	0/15	NR	13/15
					FRCR imp	15	3/15	1/15	NR	11/15
					BL	15	1/15	1/15	NR	13/15
Tayaran, 2018 [16]	Randomized Clinical Trial	40	4–9	18	CL	20	0/20	1/20	NR	19/20
		18/22			BL	20	4/20	0/20	NR	16/20
KamOOal, 2017 [20]	Randomized Clinical Trial	15	5–7	12	FRCR	15	1/15	0/15	0/15	14/15
					BL	15	2/15	0/15	0/15	13/15
Quedeimat, 2015 [14]	Randomized Clinical Trial	36	3.4–6.3	6	CL	18	1/18	3/18	0/15	14/18
		16/20			BL	18	13/18	1/18	0/15	4/18
Abdulhameed, 2014 [15]	Randomized Clinical Trial	45 26/19	4–7	12	BL	15	1/15	1/15	0/15	13/15
					DB	15	4/15	0/15	4/15	7/15
					S	15	0/15	4/15	8/15	3/15
Garg, A, 2014 [18]	Observational	30	5–8	6	FRCR	30	9/30	2/30	0/30	19/30
					BL	30	14/30	2/30	1/30	13/30
Setia, 2014 [19]	Observational	45	4–9	9	BL	15	NR	NR	NR	11/15
					Custom	15	NR	NR	NR	11/15
					BL FRCR	15	NR	NR	NR	5/15

BL: band–loop; CL: crown–loop; FRCR: fiber-reinforced composite resin.

**Table 2:** Quality for RCTs using the PEDro scale.

Items	Tyagi 2021 [21]	Potgieter 2018 [22]	Mittal 2018 [23]	Tayaran 2018 [16]	Kamal 2017 [20]	Quedeimat 2015 [14]	Abdulhameed 2014 [15]
The selection criteria were specified	**X**	**X**	**X**	**X**	**X**	**X**	**X**
Subjects were randomly assigned to groups	**O**	**O**	**O**	**O**	**O**	**O**	**O**
The assignment was hidden	**O**	**O**	**O**	**O**	**O**	**O**	**O**
The groups were similar at baseline in relation to the most important prognostic indicator	**X**	**X**	**X**	**X**	**X**	**X**	**X**
All subjects were blinded	**O**	**O**	**O**	**O**	**O**	**O**	**O**
All therapists who administered the therapy were blinded	**O**	**O**	**O**	**O**	**O**	**O**	**O**
All assessors who measured at least one key outcome were blinded	**O**	**O**	**O**	**O**	**O**	**O**	**O**
Measures of at least one of the key outcomes were obtained from more than 85% of the subjects initially assigned to the groups	**X**	**X**	**X**	**X**	**X**	**X**	**X**
Results were presented for all subjects who received treatment, were assigned to the control group, or where this was not possible, data for at least one key outcome were analyzed by “intention to treat”	**X**	**X**	**X**	**X**	**X**	**X**	**X**
Results of statistical comparisons between groups were reported for at least one key outcome	**X**	**X**	**X**	**X**	**X**	**X**	**X**
The study provides point and variability measures for at least one key outcome	**X**	**X**	**X**	**X**	**X**	**X**	**X**
**Total**	**6/11**	**6/11**	**6/11**	**6/11**	**6/11**	**6/11**	**6/11**

**X**: yes; **O**: no.

**Table 3:** NIH quality assessment tool for observational studies.

Items	Abdin, 2024 [13]	Tahririan, 2019 [17]	Garg, 2014 [18]	Setia, 2014 [19]
Research question	**X**	**X**	**X**	**X**
Study participants	**X**	**X**	**X**	**X**
Participation rate	**O**	**X**	**X**	**X**
Population	**X**	**X**	**X**	**X**
Sample justification	**X**	**X**	**X**	**X**
Timeframe	**X**	**X**	**X**	**X**
Different levels	**O**	**O**	**O**	**O**
Exposure measures	**X**	**X**	**X**	**X**
Outcome measure	**X**	**X**	**X**	**X**
Assessors blinded	**O**	**O**	**O**	**O**
Loss to follow-up	**X**	**X**	**X**	**X**
Potential confounding variables	**O**	**O**	**O**	**O**
**Quality rating**	**G**	**G**	**G**	**G**

**X**: yes; **O**: no; G: good quality.

## Data Availability

Not applicable.
